# Combining Realist approaches and Normalization Process Theory to understand implementation: a systematic review

**DOI:** 10.1186/s43058-021-00172-3

**Published:** 2021-06-26

**Authors:** Sonia Michelle Dalkin, Rebecca J. L. Hardwick, Catherine A. Haighton, Tracy L. Finch

**Affiliations:** 1 Faculty of Health and Life Sciences, Northumbria University, Newcastle upon Tyne, UK; 2 Fuse (The Centre for Translational Research in Public Health), Newcastle upon Tyne, UK; 3 Faculty of Health, University of Plymouth, Plymouth, UK

**Keywords:** Realist, Normalization Process Theory, Systematic review

## Abstract

**Background:**

Realist approaches and Normalization Process Theory (NPT) have both gained significant traction in implementation research over the past 10 years. The aim of this study was therefore to explore how the approaches are combined to understand problems of implementation, to determine the degree of complementarity of the two approaches and to provide practical approaches for using them together.

**Methods:**

Systematic review of research studies combining Realist and NPT approaches. Realist methodology is concerned with understanding and explaining causation, that is, how and why policies, programmes and interventions achieve their effects. NPT is a theory of implementation that explains how practices become normalised. Databases searched (January 2020) were ASSIA, CINAHL, Health Research Premium Collection via Proquest (Family Health Database, Health & Medical Collection, Health Management Database, MEDLINE, Nursing & Allied Health Database, Psychology Database, Public Health Database) and PsycARTICLES. Studies were included if the author(s) stated they used both approaches: a scientific Realist perspective applying the principles of Pawson and Tilley’s Realist Evaluation or Pawson’s Realist Synthesis and Normalization Process Theory either solely or in addition to other theories. Two authors screened records; discrepancies were reviewed by a third screener. Data was extracted by three members of the team and a narrative synthesis was undertaken.

**Results:**

Of 245 total records identified, 223 unique records were screened and 39 full-text papers were reviewed, identifying twelve papers for inclusion in the review. These papers represented eight different studies. Extent and methods of integration of the approaches varied. In most studies (6/8), Realist approaches were the main driver. NPT was mostly used to enhance the explanatory power of Realist analyses, informing development of elements of Contexts, Mechanisms and Outcomes (a common heuristic in realist work). Authors’ reflections on the integration of NPT and Realist approaches were limited.

**Conclusions:**

Using Realist and NPT approaches in combination can add explanatory power for understanding the implementation of interventions and programmes. Attention to detailed reporting on methods and analytical process when combining approaches, and appraisal of theoretical and practical utility is advised for advancing knowledge of applying these approaches in research.

**Systematic review registration:**

Not registered.

Contributions to the literature

- Realist approaches (theory-driven methods that focus on explaining the mechanisms of action that underlie complex interventions) and Normalization Process Theory (a sociological theory used to understand the dynamics of implementing, embedding and integrating complex interventions) are increasingly being combined.
- However, Realist approaches were the main methodological framework and Normalization Process Theory was mostly used to enhance the explanatory power of Realist analyses.
- Whilst using Realist and Normalization Process Theory approaches in combination can add explanatory power for understanding the implementation of interventions, attention to detailed reporting and appraisal of utility is advised for advancing knowledge of applying theory in research.

## Background

Realist approaches to research and evaluation are theory-driven [1, 2], focussing on explaining the mechanisms of action that underlie complex programmes or interventions. An appreciation of the complex social reality inherent in the programme under investigation is required to seek the theories that explain why interventions are successful in some instances but not in others [3]. In both Realist evaluation and synthesis methods, the process begins with the development of a causal assertion of initial programme theories, representing conjectured Context–Mechanism–Outcome configurations (CMO) [2, 4] (see Table 1 for an example). The proposed CMO configuration acts as a heuristic, reminding the Realist researcher that in order to understand complex interventions and their implementation, we need to consider causal powers of mechanisms and enabling context.

**Table 1:** A CMO of a prison rehabilitation programme, adapted from Pawson and Tilley [2], p113

Context	+	Mechanism	=	Outcome
Prisoners with little or no previous education with a growing string of convictions – representing a ‘disadvantaged’ background		Modest levels of engagement and success with the progam trigger ‘rehabilitation’ process in which the inmate experiences self-realization and social acceptability (for the first time)		Lowest levels of reconviction as compared with statistical norm for such inmates.

Realist approaches can also involve a ‘to and fro’ between the initial programme theory and more substantive (or formal) theory, that is, existing theories within particular disciplines (e.g. Third Space theory, Capabilities model, Normalization Process Theory) [5].

Research should move automatically from the new, concrete situation to be studied and out to a familiar abstract framework of necessary relationships and back to the then, not quite so new, concrete programme to be studied in more detail. This double movement concrete to abstract, to concrete, provides the glue, the bridgehead, the source of continuity between inquiries [6].

Substantive theories are used to further understand interventions, for example, theories from implementation science could help to explain why an intervention has not stood the test of time. As outlined by Pawson [6], evaluation to date has been less focused on developing abstract sets of concepts that explain how ‘families’ of policies, programmes or interventions achieve their variable outcomes. Instead, the focus has been on identifying inputs, outputs and outcomes related to *specific* programmes. However, more could be achieved as a research community should we use ‘mutual learning’ [6] by drawing on substantive theories; in doing so, the theory underlying interventions in a particular domain can be transferable to other domains, topics or fields of services.

One such substantive theory, specific to the domain of implementation science, is Normalization Process Theory. Normalization Process Theory (NPT) is a sociological theory that we can use to understand the dynamics of implementing, embedding and integrating some new technology or complex intervention [7]. As an Action Theory, NPT is concerned with explaining what people do (rather than their attitudes or beliefs), and with reference to the social and organisational contexts in which those actions take place. It is intended as a theory-based approach that can help managers, clinicians and researchers to understand—and ultimately shape—processes involved in implementing, embedding and integrating service innovations into practice [8]. NPT proposes four constructs that represent different kinds of work that people do around implementing a new practice, each representing a form of social action: Coherence, Cognitive Participation, Collective Action and Reflexive Monitoring [7]. Accordingly, NPT can help researchers to think about problems of implementation by asking questions like ‘what is the work involved here?’ (Coherence), ‘who does that work?’ (Cognitive Participation), ‘how do those involved do the work to make it happen in practice?’ (Collective Action) and ‘how do they assess and respond to the impacts of their work?’ (Reflexive Monitoring). By asking these questions, we can identify problems of implementation that we might not have anticipated—and come up with solutions to make things work more smoothly for those involved.

As defined by RAMESES II [9], NPT can be considered a substantive theory, that is, a theory that operates across and within different domains or disciplines. However, NPT is not clearly a Realist theory in the tradition or school of Pawson and Tilley [2], sometimes referred to as Scientific Realism [10], as it does not ‘label’ the constructs within it as contexts, mechanisms or outcomes; it makes no claims to the constructs within it as having causal powers for certain outcomes that are only generated under certain contexts.

NPT has now been in use since 2007. As the publication of an increasing number of studies drawing on both approaches has become evident, it is timely to understand how this substantive theory is used in combination with a Realist approach and what value this combination can add to explaining complex social interventions. A recent review of NPT studies [11] included only studies in which NPT was the *primary* theoretical framework used in the study. It did not investigate how NPT was used in combination with other theoretical approaches, such as Scientific Realism. Furthermore, as Realist approaches utilise substantive theory to enhance the explanatory endeavour, making explicit how this is achieved through examination of a highly cited substantive theory, in the popular field of implementation science, is of importance to enhance learning and inform future practice in both use of NPT and Realist approaches, as well as how Realist work can use substantive theory. Finally, the commonalities and differences between NPT and Realist approaches can also be extrapolated, for example, are the mechanisms as described in NPT translatable (and in what ways) to the mechanisms used in Realist approaches?

The aim of this research was therefore to systematically review the combined use of Realist approaches and NPT. Specific objectives were to review ways in which the two approaches have been used: what is the dominant methodological driver and why? To understand the complementarity of NPT and Realist approaches; in what ways is NPT an inherently Realist theory? To provide practical recommendations for using the approaches together; building on the work that has already been completed, how can we improve the use of NPT and Realist approaches?

## Methods

This research was based on publicly available published data and therefore did not require research ethics committee approval.

To understand the combined use of Realist approaches and Normalization Process Theory, a systematic review was conducted and reported according to the Preferred Reporting Items for Systematic Reviews and Meta-Analyses (PRISMA) [12].

This review was based on an explicit, pre-specified protocol; however, as this review did not include a health-related outcome, it was not eligible for registration with PROSPERO the international database of prospectively registered systematic reviews in health and social care, welfare, public health, education, crime, justice and international development.

### Search strategy

A search was conducted in January 2020, using terms based on the concepts of Realist Research and Normalization Process Theory using the text string: “Normali?ation Process Theory” OR “Normali?ation Process Model” OR NPT OR NPM AND Realist OR Realism OR “Pawson and Tilley” in any field. The following databases were searched: ASSIA, CINAHL, Health Research Premium Collection via Proquest (Family Health Database, Health & Medical Collection, Health Management Database, MEDLINE, Nursing & Allied Health Database, Psychology Database, Public Health Database) and PsycARTICLES. Databases provided authoritative and comprehensive coverage of the literature relating to social sciences, sociology, economics, politics, psychology, social work, nursing, health, allied health, human resource management, consumer behaviour and organisational change. Materials from over 950 journals are included in CINAHL whilst MEDLINE contains millions of citations, derived from thousands of biomedical and life science journals, extending back to 1946, annual input now exceeds 700,000 citations. Other sources used to identify relevant papers were the reference lists of included studies, google scholar and contact with experts in the field. EndNote reference manager was used to store retrieved references and remove duplicate entries.

### Inclusion and exclusion criteria

Studies were included if the author(s) stated they used both approaches: a scientific Realist perspective applying the principles of Pawson and Tilley’s Realist Evaluation [2] or Pawson’s Realist Synthesis [13] and Normalization Process Theory [7] either solely or in addition to other theories (as is usual in Realist approaches). A simple reference to both theories within a paper did not make it eligible for inclusion; only papers where both approaches were utilised to some extent were included. Studies carried out worldwide were included; however, due to resource limitations, only those reported in the English language were included. Any empirical study design (including primary research and syntheses) published in the peer-reviewed literature from 2007 onwards (when Normalization Process Theory was first developed) that met the inclusion criteria were included. Book reviews, editorials, opinion pieces, conference abstracts and protocols were excluded.

Studies that did not combine the use of Realist approaches (evaluation, synthesis and research) and Normalization Process Theory, and studies that used Critical Realism, as opposed to Scientific Realism [13] were excluded. Judgement on this was by consensus amongst all authors, with a minimal threshold for inclusion being that both approaches were used in the same study. The differences between Critical Realism and Scientific Realism, as it has come to be known, are outlined in a robust exchange elsewhere [13, 14]. This exclusion criterion was deemed necessary for two reasons: so that the study could focus on how Normalization Process Theory worked as a substantive theory in Realist inquiry and secondly because a key distinction between Scientific Realism and Critical Realism is the extent to which realism is mainly a matter for philosophy and philosophers (Critical Realism) or realism as a philosophy that is inextricably linked to the practice of research and evaluation (Scientific Realism). The implication being that we were interested in studies which used realism in the practice of doing research, rather than realism being a more remote abstract philosophical idea.

### Data extraction (selecting and coding)

An initial screening of titles and abstracts against the inclusion criteria was carried out to identify potentially relevant papers followed by a screening of the full papers identified as possibly relevant in the initial screening. The screening was carried out by two of the authors independently (CH/SD) with any differences resolved by discussion or involvement of a third author as necessary (TF). Data was extracted using a structured data extraction table developed and applied by three of the authors (SD/TF/RH). Data extraction was checked by CH.

### Quality appraisal and data analysis

This review was aimed at understanding the use of Realist evaluation and NPT together, rather than synthesising evidence of intervention effectiveness; therefore, tools such as the Critical Appraisal Skills Programme were deemed inappropriate for assessing the studies in this review. Instead, studies were checked to establish if they stated that RAMESES guidance for Realist evaluation methodology had been followed. Lack of reported use of RAMESES guidance was not used as an exclusion criterion. All papers were reviewed for their application of the two approaches by co-authors with expertise in Realist evaluation (SD/RH) and NPT (TF). Due to the aim of this review and the type of data extracted, neither a meta- (a specific statistical strategy for assembling quantitative results of several studies into a single estimate) nor a thematic (an approach to synthesis that integrates findings usually in the form of direct quotations from multiple qualitative studies) analysis was appropriate; therefore, a narrative synthesis was employed.

## Findings

### Brief description of studies

**Fig. 1:**
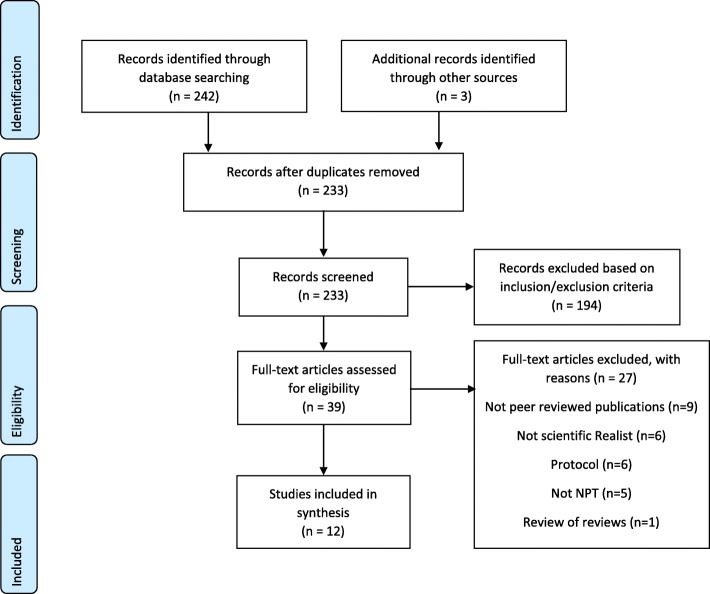
PRISMA flow diagram

Twelve papers [8, 15–25], reporting eight studies, were identified which met the inclusion criteria (Fig. 1). Table 2 provides a summary of these papers which were all published between 2015 and 2020. Three papers reported on the RAPPORT study (ReseArch with Patient and Public invOlvement: a RealisT evaluation) [16, 18, 20] which utilised a three-stage Realist evaluation drawing on Normalization Process Theory to understand how far patient and public involvement was embedded within healthcare research. Three other papers [19, 21, 24] were based on a Realist evaluation on the sustainability of Lean in paediatric healthcare. All other papers reported individual studies [8, 15, 17, 22, 23, 25].

**Table 2:** Summary of studies

Author and date	Setting and country	Study type	Aim	Intervention description	Participants	RAMESES
1. Gillespie et al. 2015 [8]	Operating rooms UK (n = 9), USA (n = 7), New Zealand (n = 2), Netherlands (n = 2), Finland (n = 2), other countries (n = 12), multinational (n = 1)	Realist synthesis	To present the evidence of implementation interventions to improve adherence to the use of safety checklists in surgery	Surgical safety checklists	35 primary studies	No
2. Goodridge et al. 2015 [15]	Healthcare organisations Canada	Qualitative interviews	To develop initial programme theory about the role of leadership in the Saskatchewan model of Lean	Lean approach to improving healthcare	27 participants (26 face to face interviews, 1 telephone interview). Key stakeholder interviews (9), stakeholder focus group (n = 47)	No
3. Wilson et al. 2015 [16] (RAPPORT study)	Healthcare research UK Stage 1 England and Wales, stages 2 and 3 four geographical regions in England	Mixed methods (scoping exercise/online survey to chief investigators/case studies/interviews and document analysis)	To understand how far patient and public involvement was embedded within healthcare research in six areas: diabetes mellitus, arthritis, cystic fibrosis, dementia, public health and learning disabilities	Patient and public involvement (PPI) in research	101 participants completed the survey, 22 case studies, 129 participants [64 researchers, 48 PPI representatives, 7 PPI co-ordinators, 10 funders/network representatives]	No
4. Tsang et al. 2016 [17]	Primary care UK (n = 8), other European countries (n = 2), North America (n = 8)	Rapid Realist review	To understand the factors enabling and constraining the implementation of CKD interventions in primary care	CKD interventions	18 interventions in primary care between 2000 and 2014	Yes
5. Howe et al. 2017 [18] (RAPPORT study)	A case study of a project on patient and public involvement in research UK	Qualitative case study Review of documentation and experiences of PPI over time	To investigate how the PPI representatives’ inputs had developed over time, key challenges and changes, and lessons learned	PPI in various forms	No specific reference to numbers of people involved in the project	No
6. Flynn et al. 2018 [19] (FLYNN study)	Healthcare organisations Canada	Realist review	To develop and refine an initial programme theory on Lean sustainability in paediatric healthcare	Lean model of healthcare quality and efficiency—philosophy and activities	11 papers—included primary research studies and published and unpublished case studies and research	Yes
7. Wilson et al. 2018 [20] (RAPPORT study)	Healthcare research England	Realist evaluation	To explore how embedded patient and public involvement is within mainstream health research following two decades of policy-driven work to underpin health research with patient and public involvement in England	Patient and public involvement (PPI) in research on six diverse topic areas: arthritis, cystic fibrosis, dementia, diabetes, intellectual and developmental disabilities and public health	22 nationally funded research projects as case studies in which to explore PPI processes and impact	No
8. Flynn et al. 2019 [21] (FLYNN study)	Healthcare organisations Canada	Qualitative Realist evaluation	To evaluate the sustainability of the Lean model in paediatric healthcare	Lean model of healthcare quality and efficiency—philosophy and activities	32 interviews with various stakeholder groups across four paediatric hospital units ‘cases’ at one acute hospital	Yes
9. Hurst et al. 2019 [22]	Acute hospital wards for care of older people UK	Mixed methods (qualitative interviews/descriptive analyses of routine data)	To evaluate the implementation of open visiting, the barriers, sustainability and impact on communication between healthcare professionals, families and carers	Open visiting—unrestricted family visiting hours	30 interviews (16 with staff; 14 with patients/relatives). 47 pre-implementation questionnaires about staff attitudes towards open visiting	Yes
10. Lewis et al. 2019 [23]	Community aged home care organisation Australia	Qualitative Realist evaluation	To evaluate the embedding of sustainable oral healthcare for older people into routine community aged care practice	Multi-level facilitation of oral health for older people resident in care homes	14 interviews, mostly staff within the care organisation plus service consumers	Yes
11 Flynn et al. 2020 [24] (FLYNN study)	Healthcare organisations Canada	Multiphase Realist investigation	To present a refined programme theory on the contextual factors and mechanisms that influence the sustainability of (Lean) management initiative in paediatric health care	Lean model of healthcare quality and efficiency—philosophy and activities	Triangulation of multiphase Realist investigation	No
12. Hashem et al. 2020 [25]	Hospice at home UK	Systematic literature review analysed using Realist logic	To determine what features of hospice at home models work best, for whom and under what circumstances	Hospice at home	49 papers reviewed, of which 34 contributed evidence to at least one of the eight theory areas	Yes

The majority of papers (9/12) in the review focused on developing a (post hoc) understanding of factors associated *with implementation, embedding and/or sustainability of* interventions that had already been implemented. These included lean approaches to healthcare [15, 19, 21, 24], public and patient involvement in healthcare research [16, 18, 20], oral healthcare for older people in community practice [23] and chronic kidney disease management in primary care [17]. Two papers focused on intervention effectiveness: Hashem et al. [25] focused on identifying mechanisms underpinning ‘what works, why and in what circumstances’ in hospice at home services for end-of-life care, and Gillespie et al. [8] conducted a realist synthesis of evidence of implementation interventions to improve adherence to the use of safety checklists in surgery. Only one study reported using the approaches to inform both (strategic) implementation as well as evaluation (understanding), of an intervention: ‘open visiting’ on wards for care of older people in the acute hospital setting [22].

### How were the approaches combined?

Most studies (6/8) included in the review used Realist evaluation as the main methodological framework [8, 15–17, 19–21, 24, 25], employing Normalization Process Theory (NPT) as a substantive theory [5]:

As we were interested in how PPI becomes embedded within clinical research, Normalization Process Theory provided an explanatory theory to inform the development of a PPI specific programme theory about how PPI has become embedded (or not) as normal practice within health research [20]

Within these studies, NPT was used at several stages, such as programme theory development, data extraction, analysis and interpretation. For example, in their Rapid Realist Review of implementation of chronic kidney disease interventions in primary care, Tsang et al. [17] described using the four NPT constructs to guide data extraction, in which:

A systematic approach to data extraction using Normalisation Process Theory (NPT) illuminated key mechanisms and contextual factors that affected implementation [17].

Wilson et al. [20] took a similar approach, using NPT as an initial coding framework, but did not refer specifically to the constructs of NPT as part of this:

We used Normalization Process Theory to provide an initial coding frame for the analysis of the interview data and documents. We followed a stepped approach to data analysis [20]

Conducting a Realist synthesis, Gillespie et al. [8] sought substantive theory (but not specifically NPT) to act as a guiding framework:

The middle-range theories we identified a priori provided a starting point in our efforts to explain what types of checklist implementation interventions work in surgery, for whom, and in what circumstances [8].

Whilst some studies used NPT explicitly to guide their programme theory development or data analysis, others reported less details about their use of NPT. The study by Goodridge et al. [15] used a Realist approach to develop an initial programme theory about the role of leadership in the Saskatchewan model of Lean. The authors stated in the abstract and introduction how NPT was used as a formal theory, chosen to help understand the initial Realist programme theory (which concerned leadership and lean initiatives). In the discussion section, NPT was referred to in terms of how it might help to understand contextual factors necessary to support lean implementation, but how NPT was operationalised as a substantive theory was not unpacked further. For example, it was not articulated specifically how the NPT framing of context worked with the initial programme theory, nor how the four constructs of NPT (coherence, cognitive participation, collective action and reflexive monitoring) related to the initial programme theory.

Two studies (out of 8 identified) [22, 23] did not use Realist approaches as the main framework for their study. Lewis et al. [23] used a ‘Realist informed approach’ to studying implementation of sustainable oral healthcare for older people into routine community aged care practice through conducting pre- and post-implementation data collection with ‘the recommended phases of the Realist evaluation cycle’. They integrated Realist approaches with NPT, by building CMO configurations within each of the four NPT construct domains, both during initial implementation of their programme and post-implementation 3 years on. Hurst et al. [22] used a more segregated approach to Realist approaches and NPT, using Realist approaches for the evaluation aspect of the study and NPT to inform and prepare for implementation of the intervention. For example, during phase 1 of their project, NPT was used to develop activities to facilitate implementation of open visiting on hospital wards that specifically targeted the NPT concepts (generating shared sense of purpose; engagement; building in processes of monitoring, etc.). Whilst NPT was stated as constituting part of the ‘overall theoretical framework’ for the study, its use in the evaluation component was more implicit than in the implementation activities, with Realist approaches driving the evaluation.

One paper (out of 12) did not explicitly operationalise a Realist approach [18]. Howe et al. [18] described embedding of patient and public involvement as part of the RAPPORT study [16, 20] but in this paper, used NPT only to focus on the embedding of patient and public involvement processes within the RAPPORT project as a case study (for which applying a Realist approach would likely have been limited). Their reference in the paper to having used ‘Realist evaluation and NPT’ was a reflection on the RAPPORT study as a whole, rather than the focus of the article itself [18].

### Overt acknowledgement of NPT constructs and/or CMO components

Several studies (3/8) did not overtly refer to or label NPT constructs in their analysis [15, 16, 18–21, 24]. Whilst some studies specified and differentiated the contexts, mechanisms and outcome components of their programme theories (e.g. Hashem et al. [25]), some studies did not explicitly label and identify the respective parts of the CMO configuration in their stated programme theories. Although there are no guidelines in either NPT or Realist approaches that state this conceptual labelling is necessary, it can help the reader to follow the analysis process from the methods through the findings. However, it is acknowledged that several authors may argue the findings are topic focused and therefore do not require such labels.

Where studies did provide NPT construct labels in reporting and/or discussing their findings [16, 17, 23, 25], it was easier for the review team to identify the methodological benefit of combining the two approaches. For example, Lewis et al. [23] began with the four NPT constructs (coherence, cognitive participation, collective action and reflexive monitoring) and applied a Realist approach analysis to develop explanations in relation to each construct, of the embedding of oral healthcare in routine practice for older people’s community care, with reference to contexts, mechanisms and outcomes.

### NPT constructs as components of CMO configurations

#### Mechanisms

Five studies framed NPT constructs as Realist mechanisms [8, 17, 19, 21, 24, 25]. Although not linking explicitly to the constructs of NPT within the paper, Gillespie et al. [8] suggest mechanisms of implementation which they align at a general level to NPT and/or Responsive Regulation Theory [26]. Underpinning this specification of mechanisms is an NPT-focused analysis that evaluates the implementation of the surgery checklist against the constructs of NPT [8]. Tsang et al. [17] and Flynn et al. [19, 21, 24], who used NPT in this way, highlighted that the constructs of NPT can be conceptualised as Realist mechanisms:

[NPT] was designed to identify and understand the processes underpinning care, through which existing interventions had become taken-for-granted or ‘normalised’ [21, 22]. These are described as coherence, cognitive participation, collective action and reflexive monitoring [21]. From a realist perspective, these can be viewed as basic mechanisms through which implementation and therefore normalisation occurs [17]

NPT offered insights into the potential mechanisms that promote or inhibit the embedding of complex interventions into routine everyday practice and the likelihood of sustainability [19]

#### Contexts and mechanisms

Two studies suggested that NPT constructs could enhance explanation across the whole Realist explanatory endeavour: context, mechanism and outcome. For example, Lewis et al. [23] used NPT as an overall framework to their study and did not align the constructs specifically to the Realist approach components but rather used the Realist evaluation cycle to achieve a higher level of explanation of how better oral health in care homes might become embedded:

When compared with other scientific paradigms, a realist approach offers this study a theoretically driven methodology with which to retrospectively and prospectively explore the interplay of Normalisation Process Theory core constructs in terms of mechanisms, context and outcomes that may have supported or hindered the embedding of the Better Oral Health in Home Care Model into routine practice (p. 34) [23]

Similarly, Hashem et al. used NPT as an overall approach to their study, and the constructs themselves as a means of developing the overall programme theories [25], but took the application of NPT constructs to a more detailed level, utilising the 16 sub-constructs. They state that:

…we found NPT a useful tool to unpick our programme theory. Using this framework, we focused our analysis on the characteristics of implementation processes and, in doing so, facilitated an understanding of contexts, social structures and processes within which hospice at home services operate in, thereby helping to understand the relationships between the mechanisms, their triggers and the effects they produce (p.19) [25]

Hashem et al. [25] commenced the development of initial programme theories using the 16 sub-constructs of NPT, to guide data extraction from the review evidence, in particular providing a rich description of context and background characteristics of the hospice at home interventions for developing initial programme theories (IPTs). Six IPTs were then developed to represent ‘key theory areas’ through mapping of the NPT sub-constructs against the National Association of Hospice at Home standards and later refined into eight proposed programme theories (CMO configurations) through a series of stakeholder workshops. The authors [25] describe having used NPT ‘as a lens’ to draw out how their programme theory areas could lead to the embedding of hospice at home services into end-of-life care service provision. It is therefore suggested here that both Lewis et al. [23] and Hashem et al. [25] have used NPT to guide the development of programme theory in a more holistic sense, and perhaps to contribute to the understanding of all three elements of the CMO configurations, rather than for developing specific components only.

#### Outcomes

One study made explicit use of NPT to inform understanding of outcomes. Wilson et al. [20] used The Economic and Social Research Council-funded web-based NPT toolkit (www.normalizationprocess.org) [27] to develop radar plots for each case study within the four NPT constructs and 16 domains. These plots are a way of visually summarising any kind of data by displaying it on multiple axes out from a central point that can be created using spreadsheet software. They have been used for representing scoring undertaken within the NPT website on ‘extent of normalization’ through ratings on items representing the 16 domains. In Wilson et al.’s study, data for the radar plots were collected at the initiation of the case study and again at 18 months, or earlier if the study had concluded:

The fuller the radar plots, the more fully embedded the PPI. Spikier plots illustrate internal variance in the degree of embeddedness. (20)

The radar plots were used alongside the individual case study Realist evaluations as ‘aides-memoires’ for the research team, helping them to understand the changes in PPI over time and supporting ongoing comparison between case studies. They were not explicitly used in CMO analysis but were used to provide illustrative before and after exemplars in the findings chapter, as a broad ‘test’ of whether PPI had become more embedded during the term of the case study.

### Study authors’ reflections on the use of Realist approaches and NPT

Most authors of the studies in this review that explicitly used both Realist and NPT together did not reflect on the value, ease or otherwise of combining the approaches [8, 15, 19–23, 25]. Some authors made passing reference that combining the two approaches was ‘useful’ [16], and/or a key strength of their study [23], but did not elaborate further. Others reflected only (and positively) on their use of a Realist approach [8, 21]. However, and perhaps one of the more ‘integrative’ studies in this review (alongside Wilson 2015 [16]), Tsang et al.’s [17] paper is an exception, providing a detailed critique of the two respective approaches (including acknowledging the risks of constraining the analytical lens by applying NPT constructs [11]). Tsang et al. [17] summarise the value of using an integrative framework built on Realist methodology and NPT:

We have established a framework to understand the complex processes surrounding implementation by integrating NPT with realist methodology to describe the individual and collective work of embedding and integrating CKD interventions into a particular context. Our methodology allowed the dissection of each intervention to identify separate components within an intervention that were well implemented and other parts that were not (p.12)

## Discussion

Twelve papers [8, 15–25], reporting eight studies, were identified which met the inclusion criteria. Although in most studies, Realist approaches were the key overaching framework, NPT was used at several stages, such as programme theory development, data extraction, analysis and interpretation. Within the analysis of these studies, often concepts from NPT were used as parts of the Context–Mechanism–Outcome configuration. No studies used the full CMO heuristic to explain their findings, instead using it to describe mechanisms or context or outcomes.

### How were the two approaches combined?

Realist approaches were the overarching framework in 6/8 studies [8, 15–17, 19–21, 24, 25], with NPT employed as an explanatory (substantive) theory [5]. This emphasis on Realist approaches as the main driver may have been due to Realist approaches providing a more prominent methodological and conceptual structure. Whilst the RAMESES guidelines have tried to avoid overly prescriptive ‘step by step’ instructions, they are grounded in Scientific Realism which means that there are core elements that researchers need to adhere to, such as being theory-driven, adhering to generative causation and using an iterative approach [9]. Alternatively, NPT was initially empirically driven as opposed to philosophically. It emerged out of three key programmes of observational/ethnographic research that studied interventions that change practice: patient/professional consultations (shared decision-making), clinical guideline development and telemedicine/telecare [7]. This empirical and practice-led development potentially now allows the researchers utilising the approach more flexibility in how they apply the theory, hence it being used as a substantive theory in most studies in the review. To some extent, this finding was to be expected as the review explicitly sought papers that had used both approaches. What has been found though is *how* NPT and realist approaches have been combined: that is, NPT is utilised as a substantive theory within a realist inquiry: NPT undertakes a supportive role in both the research design and explaining the findings within realist studies.

NPT was used in several studies to inform the components of CMO configuration, but only one study (Wilson et al. [20]) reported using NPT to describe outcomes. This prompted discussion around the usefulness of NPT radar plots when used in Realist approaches; radar plots are a ‘measure’ of ‘implementation outcome’, which could potentially help in understanding the outcomes component of the CMO configuration. However, the measure can be considered quite crude and a more sophisticated survey tool is now available—NoMAD [28]—that meets measurement property standards. A common approach in Realist evaluation is to begin by exploring patterns of variable outcomes and then track back to understand and theorise on the associated context and mechanisms at play (a form of retroductive reasoning) [10]. This can be achieved using qualitative or quantitative data. The use of radar plots or NOMAD may be one way of indicating implementation outcomes which can then be further explained in line with generative causation using qualitative approaches in the programme theory testing process.

### Is NPT inherently a Realist theory?

The review has indicated that NPT can aid in the Realist explanatory endeavour; it highlights how NPT can enhance the explanation of the implementation process under study, but also potentially increase portability of findings through reference back to general mechanisms that transcend the programme theories of specific studies. Whilst study authors [17, 19, 20, 24] described their use of NPT concepts to develop understanding of mechanisms for CMO configurations, they did not themselves provide this level of reflection. Yet as the Realism of Pawson and Tilley is more a way to make sense of theory and data, NPT in and of itself is not an inherently Realist theory, but most of the studies included in the review ‘superimpose’ the analytic heuristic components of context, mechanism and outcome upon it. However, often, detail was lacking around how and why a certain NPT construct (e.g. collective action) was conceptualised as a context, mechanism or outcome. This could be due to a plethora of reasons which are beyond lack of analysis, such as journal word limitations, or journal focus (on topic as opposed to methods). What became evident whilst looking at the conceptualisation of NPT constructs as mechanisms was the difference between the definition of mechanisms from NPT and Realist perspectives. NPT would class the four main concepts within the theory as (‘generative’) mechanisms [7]. These constructs focus on the actions of participants or groups to achieve (or not) normalization; NPT explains implementation and embedding of practices by providing a lens on how the ‘work’ is achieved by those involved in it. Using a Realist ontology, the four concepts can be conceived as being within the realm of the empirical. The empirical is a realm that is observable, perceptible, experiential, describable and often measurable [10]. Those involved in the conceptualisation of NPT would see the ‘mechanisms’ as enabling change in that they represent different kinds of work that need to happen to enable normalization or embedding of a new practice. However, Realists would conceptualise mechanisms at the actual or real level of reality, not the empirical, as they are non-observable (but proxies can sometimes be found for their measurement) [10]. Whilst NPT is focused on the actions people or groups take, Realist approaches should also aim to uncover the non-visible drivers behind those actions, the how and why, i.e. the generative mechanisms. This is especially true because Realists assert that reasoning (including attitudes, beliefs, prejudices) guides what participants do in terms of the action they take in response to interventions. However, this does not make NPT and Realist approaches incompatible, we argue that it actually makes them complimentary. NPT can direct the Realist evaluator or reviewer to actions occurring in the empirical, but it is up to the Realist researcher to then take the next step in delving deeper to further understand the generative mechanisms which cause those actions, and how these are influenced by the study contexts, and in doing so enhance the explanatory nature of their work.

### How can the use of NPT and Realist approaches be improved?

The discussion above begs the question of whether NPT requires further ontological development, to which we believe the answer is no. NPT has been a very successful and useful substantive theory [11, 29] which has been successfully applied across several different topics, disciplines and philosophical orientations. The use of substantive theories in Realist research should provide a lens through which it is possible to make better sense of the data. NPT did not set out to be a (scientific) Realist theory, from the school of Pawson and Tilley [2] and indeed few substantive theories follow that tradition. However, as substantive theory for understanding the implementation of evidence-based interventions, NPT sits well within a Realist framing. It helps Realists to explain further the findings of their evaluation and to avoid reinventing the theory wheel [30]. However, Realists need to exercise caution when using theory (such as NPT) and give thought to the compatibility at an ontological level (and make adjustments to their analysis as necessary), in order to get the most benefit and appropriate ontological depth from their analysis.

### Limitations

Authors’ lack of reflection on the utility of applying particular theoretical approaches is a limitation that is not unique to this review [11]. Theoretical advancement would be enhanced by greater engagement of authors in reflective discussion; however, it is acknowledged that often space constraints in journal guidelines mean authors have to prioritise what to include.

Issues related to time and funding constraints meant aspects of the review could be deemed less rigorous; for example, translation services were not available and therefore the searches were limited to include only studies in the English language. In addition, there is a risk that CMO was used within studies but not attributed to the Realist approach and therefore some papers could potentially have been missed. However, screening was carried out by two of the authors independently (CH/SD) with any differences resolved by discussion or involvement of a third author as necessary (TF). Furthermore, although data were extracted using a structured data extraction table by three of the authors (SD/TF/RH) and was checked by CH, the expertise of the three academics varied, two had expertise in Realist approaches (RH/SMD) and one in NPT (TF). To account for this, where issues of uncertainty arose, a person with expertise in the other approach would also read the paper and provide comment.

The review was limited to only studies using NPT and not all implementation theories. NPT is now one of the most highly cited and utilised Implementation theories in applied health research: the primary theoretical publication [7] alone has been cited over 1000 times, and the 108 studies reviewed in 2018 [11] represented a focused selection of studies that used NPT as the only theoretical approach (many studies use multiple approaches). As other implementation science approaches gain increasing application in research to evaluate interventions and programmes (see, for example, Kirk et al.’s [31] review of the application of the Consolidated Framework for Implementation Research (CFIR)), learning from this review can be extrapolated to other implementation theories and can also inform the use of other substantive theories in general when engaging in a Realist process of analysis.

## Conclusion

Realist approaches and NPT have both gained significant traction in the past 10 years, especially in healthcare. We believe this is due to the increased explanatory potential they offer academics, practice professionals and policy makers. There is no definitive ‘correct’ way to combine the two approaches, but most academics to date have used Realist approaches as the overarching framework, then used NPT to enhance their explanatory endeavour, utilising aspects of the key Realist heuristic alongside NPT concepts. Realist researchers utilising NPT, or indeed any substantive theory in any particular paradigm, need to consider ontological depth. Furthermore, NPT or any theory should not be used as a tokenistic nod to inclusion of theory which can be desired by funders or peer-reviewed journals but should be considered and explicit. We encourage academics to include as much detail as possible on the process of analysis when combining approaches, and to offer their own reflections on their applications of theory in their research, as this will aid other researchers and facilitate learning.

## Data Availability

Not applicable.
